# Unravelling the Complexity of Plant Defense Induced by a Simultaneous and Sequential Mite and Aphid Infestation

**DOI:** 10.3390/ijms20040806

**Published:** 2019-02-13

**Authors:** Małgorzata Kiełkiewicz, Anna Barczak-Brzyżek, Barbara Karpińska, Marcin Filipecki

**Affiliations:** 1 Department of Applied Entomology, Warsaw University of Life Sciences—SGGW, 02-776 Warsaw, Poland; malgorzata_kielkiewicz@sggw.pl; 2 Department of Plant Genetics, Breeding and Biotechnology, Warsaw University of Life Sciences—SGGW, 02-776 Warsaw, Poland; barczak.annak@gmail.com; 3 School of Biosciences, College of Life and Environmental Sciences, University of Birmingham, Birmingham B15 2TT, UK; B.Karpinska@leeds.ac.uk

**Keywords:** *Myzus persicae*, *Tetranychus urticae*, co-infestation, local and systemic responses, reproduction performance

## Abstract

In natural and agricultural conditions, plants are attacked by a community of herbivores, including aphids and mites. The green peach aphid and the two-spotted spider mite, both economically important pests, may share the same plant. Therefore, an important question arises as to how plants integrate signals induced by dual herbivore attack into the optimal defensive response. We showed that regardless of which attacker was first, 24 h of infestation allowed for efficient priming of the *Arabidopsis* defense, which decreased the reproductive performance of one of the subsequent herbivores. The expression analysis of several defense-related genes demonstrated that the individual impact of mite and aphid feeding spread systematically, engaging the salicylic acid (SA) and jasmonic acid (JA) signaling pathways. Interestingly, aphids feeding on the systemic leaf of the plant simultaneously attacked by mites, efficiently reduced the magnitude of the SA and JA activation, whereas mites feeding remotely increased the aphid-induced SA marker gene expression, while the JA-dependent response was completely abolished. We also indicated that the weaker performance of mites and aphids in double infestation essays might be attributed to aliphatic glucosinolates. Our report is the first to provide molecular data on signaling cross-talk when representatives of two distinct taxonomical classes within the phylum *Arthropoda* co-infest the same plant.

## 1. Introduction 

In natural and agricultural conditions, plants are often attacked by multiple herbivores. Similar to single infestations, plant’s defense responses can modulate diverse pest performance with a broad to narrow specificity spectrum [1,2,3,4,5,6,7,8]. Induced responses are triggered by elicitors such as herbivore-associated molecular patterns (HAMPs) and damage-associated molecular patterns (DAMPs) [9,10]. HAMPs are herbivore-derived molecules coming from the invaders’ oral or ovipositor secretions, while DAMPs are released by the host plant undergoing necrosis and are related to the mode of herbivore feeding and the range of tissue damage at the feeding site [11]. 

For a given host plant, herbivores representing similar feeding strategies, salivary composition, type and range of wounding, etc., trigger a systemic acquired resistance (SAR), which by phytohormonal signaling [jasmonic acid (JA)/ethylene (ET), salicylic acid (SA), abscisic acid (ABA)] spreads to non-infested, distal organs of the plant [12]. Consequently, a herbivore-infested plant benefits from a type of nonspecific and systemic defense signal. Alternatively, the attacker can allocate plant resources for its own benefit, which may reduce the other attacker’s performance [13,14,15,16]. On the other hand, a herbivore is able to impact the host plant by systemically suppressing the most effective defense signaling pathways, which may help the subsequent invader [13,14,15]. The knowledge of the mechanisms of such remote effects of herbivory is still limited. For example, in aphid-infested *Arabidopsis* leaves, a huge defense signaling gene expression reprograming was shown compared to that in non-infested control leaves from the same plant [17]. Similarly, systemic changes in defense signaling in the non-infested leaves of the mite-infested host-plants can prepare the plant for the attack of the next invader. The growing knowledge of systemic effects of the mentioned herbivores opens another, mostly unexplored field—the defense signaling crosstalk and integration with various biotic and abiotic stresses.

The green peach aphid, GPA (*Myzus persicae* Sulzer, 1776; Hemiptera: Aphididae) and the two-spotted spider mite, TSSM (*Tetranychus urticae* Koch, 1836; Prostigmata: Tetranychidae) are economically important arthropod herbivorous pests attacking a wide range of host plants and inflicting serious damage and crop yield losses. They may either attack the plant individually or share the same plant. Although mites and aphids are piercing-sucking herbivores, they suck up distinctly from the host-plant. TSSM feeds mostly from the cells of mesophyll tissues [18], whereas GPA feeds from phloem [19,20].

TSSM, as a generalist, is able to feed on a wide range of host-plants, even on those with a high level of xenobiotics [21,22]. It probably manipulates plant defense (e.g., by suppression, induction or counteraction) [13,14,15] using effector-like proteins occurring in its saliva [23] and digestive proteases in the midgut [24]. In response to mite saliva and chelicerae wounding, the mite-infested host-plant defends itself by activating the JA-, ET-, SA- and ABA-dependent signal-transduction pathways [13,25,26,27,28,29,30,31,32]. 

GPA is a generalist hemipteran herbivore that sucks up the phloem sap of several wild and crop plants. Its mode of feeding with intracellularly penetrating stylet is very sophisticated and results in the activation of specific set of signaling pathways. It was shown that in *Arabidopsis thaliana* (L.) Heynh. following GPA feeding, the expression of the *PR1* gene (marker of SA-signaling) was induced [33,34]. However, from more recent work [17], we can learn that in both local and distal leaves of *Arabidopsis*, beside the SA-signaling, ET- and ABA- signalings were also activated in response to GPA, whereas JA-signaling was barely noticeable. This can result either from a SA-JA antagonism or the presence of effector proteins in GPA saliva [15]. 

Although recent extensive studies have improved our understanding of the interactions between *Arabidopsis* and GPA, as well as TSSM when the plant is individually infested [28,32,35,36], still little is known on the host-plant defense signalings when the plant is exposed to both pests. So far, it has been shown that dual aphids-mites infestation affects the biochemistry of tomato [3] and is regulated indirectly by tending ants [1], but still, there is no data about the molecular background of the abovementioned interaction. 

In light of the engagement of distinct signaling pathways in the defense responses of mite-infested and aphid-infested host-plants, we assessed how the presence of each of the abovementioned attackers influences the reproduction performance of the other sharing the same host plant. We also speculate on how the plant is able to integrate signals into optimal defensive responses. Pest-induced defense, along with the constitutive plant defense are one of the crucial components of integrated pest management (IPM). Therefore, better understanding of the interplay between signaling pathways ensuring the effective regulation of defensive responses within damaged and undamaged systemic organs is immensely important.

## 2. Results 

### 2.1. TSSM and GPA Reproductive Performance

**Figure 1:**
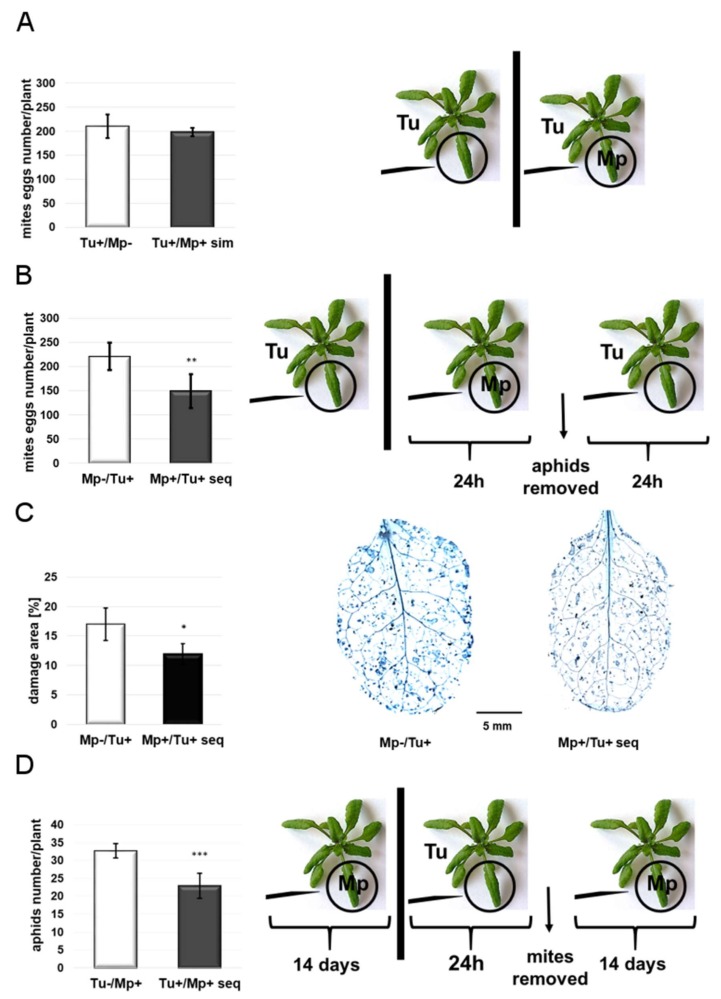
TSSM (Tu) and GPA (Mp) reproductive performance. (**A**) TSSM oviposition rate expressed as the number of mite eggs x plant^-1^ after simultaneous plants infestation with GPA and TSSM (±SD; *n* = 6). (**B**) TSSM oviposition rate expressed as the number of eggs × plant^−1^ after sequential infestation of plants by GPA and TSSM (±SD; *n* = 6). (**C**) Mite-leaf damage visualization by TB staining assessment -extent of damage assessed by the percentage of damaged leaf area (±SD; *n* = 3). Scale bar indicates magnification. (**D**) The effect of TSSM infestation on GPA reproduction (±SD; *n* = 9). Asterisks indicate significant differences (the two-tailed—*t*-test) at *p*-values of <0.05 *; <0.01 **; <0.001 ***).

To assess the influence of one attacker on the reproduction of the other sharing the same *Arabidopsis* plant, simultaneous and sequential infestations were carried out. In the case of simultaneous infestation, when TSSM was transferred onto *Arabidopsis* rosette leaves with only a few min delay after GPA had been caged on one of the leaves of the rosette, the presence of GPA had no impact on the oviposition of TSSM (Figure 1A). However, in the case of sequential infestation, 24 h delay of TSSM infestation relative to GPA infestation was sufficient to trigger defense processes resulting in significant decrease of the TSSM oviposition rate (Figure 1B). The mite performance reduction was accompanied by a significantly lower mite feeding activity measured as leaf damage area (Figure 1C). Thus, the results show that GPA-induced defense responses are effective against TSSM. 

In our next experiment assessing the effect of short-time TSSM infestation on GPA reproduction, we took into consideration a two-week period of aphid development sufficient for GPA to produce offspring and for the offspring to mature. In this way, we documented that compared to the control (plants non-infested by TSSM), a significantly smaller GPA colony developed on those *Arabidopsis* rosettes that had been previously infested with TSSM for 24 hours (Figure 1D). This clearly shows that TSSM-induced plant defense is effective against GPA. Together, GPA and TSSM interact with each other negatively only when they occur sequentially. 

### 2.2. Gene Expression Analysis 

#### 2.2.1. Local Responses to Single TSSM and GPA Infestation

**Figure 2:**
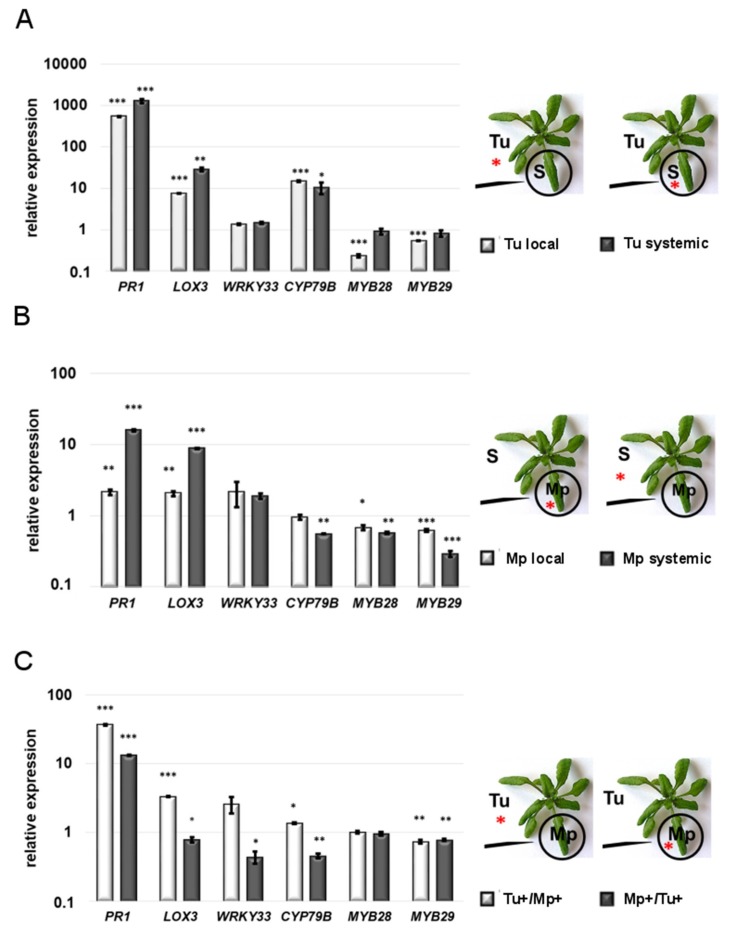
The comparison of defense-related gene expression in local and systemic *Arabidopsis* leaves upon single and simultaneous feeding of TSSM (Tu) and GPA (Mp), normalized against *ACT2* mRNA level. (**A**) Single TSSM infestation. (**B**) Single GPA infestation. (**C**) Simultaneous TSSM and GPA infestation. S—systemic leaf. Red asterisks indicate leaves collected for gene expression assessment. Error bars represent standard deviation and black asterisks—*, ** and *** represent significant differences at *p*-values of <0.05, <0.01 and <0.001, respectively (*n* = 3). The values of the relative gene expression fold changes were listed in Supplementary material (Table S1).

To better elucidate the mechanism of local and systemic plant defense responses following single, simultaneous and sequential GPA and TSSM infestation, we measured the expression of genes related to the JA-, SA- signalings and glucosinolate biosynthesis. Unsurprisingly, compared to the non-infested control, in individually mite-infested *Arabidopsis* leaves (for 24 h), the *PR1* gene related to SA signaling and the *LOX3* gene involved in the JA biosynthesis (lipoxygenase pathway) [9] showed very strong activation, reaching levels of 542- and 7-fold increase, respectively (Figure 2A and Table S1). Interestingly, we did not observe a significant change in the expression of the *WRKY33* gene which is regarded as a regulator of the SA-JA cross-talk [37,38]. Similar to the SA and JA markers, the *CYP79B* gene involved in indole-glucosinolates (IGs) biosynthesis was over 14-fold up-regulated. In contrast, the expressions of the *MYB28* and *MYB29* genes coding for transcription factors regulating the aliphatic glucosinolates (AGs) biosynthesis [39] were significantly reduced.

After 24 h of individual GPA feeding on an *Arabidopsis* leaf, the *PR1* and *LOX3* genes showed also significant 2-fold activation, which was clearly much weaker than in the case of the plant response to individual TSSM feeding (Figure 2A,B). This change, apparently reflecting attacker-specific the SA/JA equilibrium, was accompanied by an approximately 2-fold up-regulation of the *WRKY33* gene. In contrast to the *Arabidopsis* response to TSSM, GPA did not activate the IGs marker—the *CYP79B* gene. On the other hand, the *MYB28* and *MYB29* genes, the markers of AGs, showed slight but statistically significant suppression as compared to the control (non-infested) plant. Thus, they respond similarly to these genes in a TSSM-infested plant.

#### 2.2.2. Systemic Responses to Single TSSM and GPA Infestation

The systemic leaf response to infestation with TSSM only were quite similar but not identical to the local response of the directly mite-infested leaf (Figure 2A,B). *PR1* and *LOX3* genes even showed a systemic up-regulation that was twice as strong as that observed in the locally mite-infested leaves; however, the *PR1*/*LOX3* ratio was similar in both the local and systemic leaves. This could be related to the expression of *WRKY33* gene, which remained unchanged. *CYP79B* gene was systemically up-regulated, similar to the level of its local activation and different from the *MYB28* and *MYB29* gene responses, which were stably expressed in the systemic leaf. 

The systemic leaf response to GPA (Figure 2B) was also stronger than that observed locally in the aphid-infested leaf—both the SA and JA signaling markers reached 16- and 9-fold increases, respectively. The systemic up-regulation of the *WRKY33* gene and down-regulation of both the *MYB28* and *MYB29* genes remained at the same level just like in the case of the locally aphid-infested leaf. Interestingly, only in the systemic leaf did the IGs marker—the *CYP79B* gene—respond to GPA by down-regulation. 

#### 2.2.3. Systemic Responses to Double Infestation

In the double infestation experiment, GPA feeding on the systemic leaf of the plant simultaneously infested with TSSM efficiently reduced the magnitude of the SA- and JA-dependent gene expression that was locally activated by a single TSSM infestation (Figure 2A,C). Specifically, the *PR1* transcript reached a 37-fold increase when GPA fed on the systemic leaf of the plant simultaneously infested with TSSM (Figure 2C), which is significant but much lower than the 542-fold up-regulation observed in the case of single TSSM infestation (TSSM local; Figure 2A). Similarly, the level of the *LOX3* transcript up-regulation induced by TSSM dropped from a 7.4-fold to a 3.3-fold level when GPA was present on the systemic leaf (Figure 2A,C). Interestingly, in the same double infestation experiment, the *WRKY33* expression did not respond significantly (Figure 2A,C). In the case of the *CYP79B* gene, its negative systemic regulation by GPA alone was inverted to the slight induction in the leaves attacked simultaneously by TSSM (Figure 2B,C). The local TSSM and systemic GPA reactivity of the *MYB28* transcript was completely abolished (Figure 2A–C), whereas the *MYB29* expression did not respond specifically to the second attacker and was consistently down-regulated (0.7-fold) as compared to the control (Figure 2A–C). 

When the gene expression was monitored in the aphid-infested leaf, systemically influenced by TSSM feeding, a 13-fold increase in the *PR1* gene expression was found, which was much higher than that observed (2.1-fold) in the GPA-alone infested leaf (Figure 2B,C). 

Considering the fact that TSSM was able to activate the *PR1* gene over 1000-fold in the systemic leaf (Figure 2A), we noted a strong suppressive effect of local GPA feeding able to temperate the *PR1* expression burst (*PR1* fold change-13; Figure 2C). This effect was accompanied by quite a substantial reduction of the *WRKY33* expression (0.44-fold), which could be attributed to different SA/JA coordination (Figure 2C). The *CYP79B* transcription was down-regulated in the aphid-infested leaf (with TSSM feeding on systemic leaves), which is clearly the other way around as observed in the systemic TSSM 29-fold up-regulation, and different from the lack of reaction to local GPA feeding (Figure 2A,C). The *MYB28* lost its reactivity in response to GPA alone feeding, while the *MYB29* was slightly down-regulated (0.77-fold), similar to its local down-regulation of the transcriptional reaction to GPA (Figure 2B,C).

## 3. Discussion 

Plants have evolved various constitutive defense mechanisms to prevent or reduce the invasion of arthropod herbivores. However, salivary effectors of pests, including TSSM and GPA, are putatively able to break the host-plant defense (including *Arabidopsis* Col-0 plant), then recruit the innate immune system(s) which limit further host-plant susceptibility and damage. Evidence from recent studies [17,32] shows that single infestation by either TSSM or GPA leads to the rapid transcriptome reprogramming; however, under each herbivore attack, the defense responses induced in *Arabidopsis* differ extensively. This suggests herbivore-specific host-plant tailoring [15]. 

Studying the reaction of the plant to more than one herbivore, understanding the systemic signals is equally as important as understanding local and immediate responses within the herbivore-infested leaf, because simultaneous attackers usually colonize and feed on the remote leaves of the same plant. Moreover, exploring the systemic responses to infestation of taxonomically distant species, we expected to shed some light onto the signal integration in shaping the final plant response. Indeed, there are only a few reports on inter-species performance modification (see references [40]. For example, Glas et al. [14] reported that TSSM could perform better on the tomato plants previously infested with another arachnid, an eriophyid mite; whereas Kessler and Baldwin [41] showed such interaction among insects—*Manduca* hornworm (*Lepidoptera*)—was suppressed by previous infestation with myrid bug (*Hemiptera*). Thus, to our best knowledge, this report is the first to show such interaction between representatives of two distinct taxonomical classes (*Arachnida* and *Insecta*) within the phylum *Arthropoda*. In the case of GPA infestation, the *Arabidopsis* transcriptome reprogramming is well documented for both the aphid-infested and systemic leaves, revealing a limited overlap among differentially expressed gene pools [17]. In contrast, there is a modest knowledge of the local and systemic discrepancy in the TSSM-induced transcriptomes, although the long-lasting systemic defense and mobile signals have been described [25,42]. 

In our studies, monitoring a limited number of defense-related marker genes upon individual GPA feeding, we observed stronger differences between the aphid-induced local and systemic responses than those between the local and systemic responses evoked by individual TSSM feeding. Such results may be explained by a strong local suppressive effect of GPA feeding, allowing for the only slight (but statistically significant) activation of the SA and JA marker genes, whereas in the systemic leaves, the SA and JA marker genes respond 4–8 times stronger. This was especially well visible for the *PR1* gene in response to individual TSSM infestation, showing several orders of magnitude higher activation, both locally and systemically. In the case of TSSM feeding, the local suppressive effect existed but to a lesser extent than in GPA-infested plant.

The discrepancies between *Arabidopsis* leaf responses to TSSM and GPA are even stronger under double infestation scenarios. The analysis of combined infestation experiments showed that GPA was a very effective local and systemic response inhibitor which flattened the SA and JA marker gene bursts locally and systemically induced by TSSM to the levels systemically induced by GPA alone or even below them. Surprisingly, such GPA-mediated, systemically reduced responses of the plant to simultaneous TSSM feeding did not improve the performance of these pests. Namely, allowing GPA-mediated systemic effects to develop for one day in the sequential infestation study—the TSSM performance decreased, which may suggest that the JA and SA signaling under a combined attack did not contribute to decreased TSSM susceptibility. A similar effect was observed when aphid performance was assessed on *Arabidopsis* plants primed by TSSM, but the mechanism could be different, because systemically feeding TSSMs were able to completely abolish local, GPA-induced JA-mediated response, while at the same time strengthening SA responses. Therefore, the lower performance of GPAs may be attributed to a stronger SA-dependent defense than in the case of a single infestation. *PR1* gene which is the most commonly used as a marker of SA signaling appeared recently to be an active player in the plant-pathogen interaction [43]. The encoded apoplastic protein PR1 has a sterol-binding activity, which may act anti-microbially [44]. The other PR1 function can be related to the embedded stress-response peptide possibly directly engaged in signaling [45].

Another mechanism which could play a role in the mutual inhibition of both attackers relies on the toxicity and deterrence of the indole- (IGs) and aliphatic-glucosinolates (AGs) or their metabolites [32,46,47]. In our study, we monitored the transcriptional activity of the selected markers of both AGs and IGs following TSSM and GPA infestation—*CYP79B* for IGs and *MYB28* and *MYB29* for AGs. Only the IG marker showed a strong local and systemic activation when *Arabidopsis* was attacked by TSSM alone, and in terms of local reaction, this is consistent with the previously presented evidence [32]. In all the other cases tested, the AG and IG markers showed either down-regulation or were not affected at all by either single and double infestations. Interestingly, while both attackers alone can efficiently suppress *MYB28*, under combined TSSM and GPA infestation, its transcription did not change, remaining at the level of the non-infested plant. Therefore, it is possible that in the case of double infestations, unchanged AGs contribute to the mutual suppression of TSSM and GPA performance. Furthermore, this indicates that IGs contributing to the JA-dependent TSSM defense in a single infestation [32,40] lose their importance when two or more attackers start to feed and inhibit the crucial defense signaling pathways. In this case, more subtle interactions could gain importance. For example, the basic level of such compounds as AGs may be sufficient to restrain attackers. Here, the JA and SA may be still involved in signal transduction and integration, but in an attacker species- and density-specific manner. Moreover, there could be another mechanism involved, such as ABA signaling, which is evolutionarily adapted to respond to coexisting multiple stresses, and its contribution to plant-pest interactions has been postulated previously [9,17,28,48,49]. This hypothesis, however, needs further experimental verification.

## 4. Materials and Methods 

### 4.1. Mite (TSSM) and Aphid (GPA) Population Rearing 

TSSM females derived from a lab colony maintained on bean plants (*Phaseolus vulgaris* L.) were used in the study [35]. Briefly, in all the bioassays and leaf damage assessments, five-day-old (hatched from 24 h old eggs) females were used. Mite-infested bean plants were grown in controlled photoperiod (day/night: 16 h/8 h), light intensity (150 µmol photons m^−2^ s^−1^) and temperature (23 °C) conditions. 

GPA (genotype G) derived from stocks collected in Scotland were kindly provided by Robert Hancock (James Hutton Institute, Invergowrie, UK). Aphid stock (>500) was maintained on six-week-old potato plants (cv. Bintje) growing in a controlled photoperiod (day/night: 16 h/8 h), at a light intensity of 150 µmol photons m^−2^ s^−1^ and at a temperature of 20 °C. Young nymphs and females at the same age were used in the study.

### 4.2. Plant Material and Experimental Design 

Three-week-old plants of *A. thaliana* (Col-0) growing in controlled photoperiod (day/night: 8 h/16 h), at light intensity of 100 µmol photons m^−2^ s^−1^ and temperature 20 °C were used in all experiments. Various ‘non-choice’ scenarios were set up to evaluate whether or not the infestation of *Arabidopsis* plant with one herbivore affects the reproductive performance of the other.

### 4.3. TSSM and GPA Single Infestation 

For the induction of local response, *Arabidopsis* rosette was infested either with 50 TSSM (Tu) females or with 60 GPA (Mp) adult females. Mites were individually placed in the middle of the rosette to let them choose leaves for feeding freely. Aphids were enclosed in transparent plexiglass cages (ø 25 mm) and one cage was clipped to one leaf of each plant. 

### 4.4. Double Infestation Experiments

#### 4.4.1. Simultaneous Infestation

Aphids (60 females enclosed in a cage) were clipped on one leaf of the *Arabidopsis* rosettes (*n* = 6) to feed for 24 h alongside 50 mite females transferred simultaneously to the remaining leaves of the rosette [Tu+/Mp+ sim]. As a control, mite-infested plants with empty cages clipped to the respective leaves were used [Tu+/Mp−]. TSSM reproductive performance—expressed as their oviposition rate—was measured in the number of mite eggs laid by all females on the *Arabidopsis* rosette. 

#### 4.4.2. Sequential Infestation

To assess whether TSSM can activate defense against GPA and vice versa, two types of experiments were carried out. In the first one, one leaf of each *Arabidopsis* (*n* = 6) was caged with 60 GPA females for 24 h. Then, aphids were manually removed from the plants and each plant was infested with 50 TSSM for 24 h [Mp+/Tu+ seq]. As a control, mite-infested plants with empty cages attached to the appropriate non-infested leaves were used [Mp−/Tu+]. TSSM reproductive performance was assessed on previously GPA-infested *Arabidopsis* plants and expressed as mite eggs laid by all females per an *Arabidopsis* rosette. 

In the second experiment, *Arabidopsis* plants (*n* = 9) were infested with 50 TSSM females per plant. Mites fed freely on the leaves. After 24 h, all mite females were manually removed using a fine paintbrush and newborn PGA nymphs (5 per plant) were caged to one of the previously mite-infested leaves [Tu+/Mp+ seq]. Plants uninfested by mites, with newborn aphid nymphs caged [Tu−/Mp+] to appropriate leaves, were used as a control. After two weeks, the reproductive GPA performance on previously TSSM-infested and control (TSSM-uninfested) leaves was assessed and expressed as the total number of aphids per plant. 

### 4.5. Leaf-Damage Assessment

Leaf damage was evaluated using the leaves from the above-described experiment on GPA/TSSM sequential infestation. Trypan Blue (TB) staining was done according to [50]. Mite-infested leaves were submerged in TB solution (0.016% TB, 8% phenol, 8% glycerol, 8% lactic acid, and 65% ethanol) in a 15 mL conical polypropylene tube, placed in a water bath at 95 °C for 2 min. and then left in staining solution overnight at room temperature. Stained leaves were cleared with 6M chloral hydrate solution diluted in water (Avantor, Gliwice, Poland) and mite-induced leaf damage was photographed with a stereomicroscope-mounted digital camera (Leica M165-FC; Leica Microsystems, Wetzlar, Germany). To quantify damage, the leaf area was outlined, the image was binarized and the damaged area was calculated using ImageJ software [51].

### 4.6. RNA Isolation and cDNA Synthesis

RNA isolation from herbivore-infested and uninfested leaves was performed using GeneMATRIX Universal RNA/miRNA Purification Kit (EURx, Gdańsk, Poland). Reverse transcription was performed using QuantiTect Reverse Transcription Kit (Qiagen, Hilden, Germany).

### 4.7. Analysis of Gene Expression

For the analysis of transcript levels, we used the Bio-Rad CFX96 Touch^TM^ System (Bio-Rad, Hercules, CA, USA) and the QuantiTect SYBR Green PCR Kit (Qiagen, Hilden, Germany) with primers listed in Supplementary material (Table S2). Relative expression levels were calculated using the expression of *ACT2* as an internal reference, according to the ΔΔ*C*t method [52]. Significant differences in the gene expression in comparison to the control were revealed using the REST tool [53].

### 4.8. Statistical Analysis

The statistical significance of the difference between means was assessed using the two-tailed Student’s test (Excel, Microsoft, Redmond, WA, USA). 

## 5. Conclusions

This report is the first to show the signaling cross-talk when representatives of two distinct taxonomical classes within the phylum *Arthropoda* co-infest the same plant. The presented data suggest that the role of SA and JA in the integration of signals triggering host-plant response to a combined attack might be less important than could be inferred from a single infestation, which gives room for other signaling mechanisms and more subtle regulations to work.

## Supplementary Materials

Supplementary materials can be found at http://www.mdpi.com/1422-0067/20/4/806/s1.
