# Supplementary material for: Unravelling the Complexity of Plant Defense Induced by a Simultaneous and Sequential Mite and Aphid Infestation
Source: Int J Mol Sci. 2019 Feb 13;20(4):806. doi: 10.3390/ijms20040806 (PMC6412847; doi:10.3390/ijms20040806)
Supplement: Supplementary file 1 [file ijms-20-00806-s001.pdf]

Supplementary material

Table S1.The value of relative gene expression changes presented on the Figure 2.

A. The value of relative gene expression changes presented on the Figure 2A.

analyzed gene	relative gene expression fold change		p-value	
	Tu+local	Tu+systemic	Tu+local	Tu+systemic
PR1	541.72	1291.76	0.0004	0.0005
LOX3	7.44	28.54	1.97232E-05	0.004
WRKY33	1.35	1.46	0.16	0.06
CYP79B	14.67	10.37	0.0005	0.04
MYB28	0.23	0.91	8.03605E-07	0.38
MYB29	0.54	0.82	0.00098	0.14

B. The value of relative gene expression changes presented on the Figure 2B.

analyzed gene	relative gene expression fold change		p-value	
	Mp+local	Mp+systemic	Mp+local	Mp+systemic
PR1	2.14	15.91	0.003	0.0003
LOX3	2.06	8.88	0.007	0.0001
WRKY33	2.16	1.90	0.29	0.06
CYP79B	0.96	0.56	0.55	0.003
MYB28	0.68	0.58	0.03	0.003
MYB29	0.62	0.29	0.0002	7.25437E-05

C. The value of relative gene expression changes presented on the Figure 2C.

analyzed gene	relative gene expression fold change		p-value	
	Tu+/Mp+	Mp+/Tu+	Tu+/Mp+	Mp+/Tu+
PR1	36.84	13.24	0.0003	9.27755E-06
LOX3	3.31	0.78	5.20216E-05	0.02
WRKY33	2.57	0.44	0.05	0.04
CYP79B	1.37	0.45	0.01	0.004
MYB28	1.01	0.95	0.95	0.58
MYB29	0.73	0.77	0.002	0.001

Supplementary material

Table S2. List of primers used in RT-qPCR analysis

locus	gene name	forward primer	reverse primer
At3g18780	*ACT2*	TTCACCACAACAGCAGAGCGGG	CGTGATGACTTGCCCATCGGGT
At2g22330	*CYP79B3*	TCTACCGATGCTTACGGGATTG	TACAAGTTCCTTAATGGTTGGTTTG
At1g17420	*LOX3*	AGCCGAGTAGAGAAGCCA	CGACCTTAGGGAGAGGAAAC
At5g61420	*MYB28*	TGTCTGATTAGGGTTGAAACGGTG	CTATGACCGACCACTTGTTGCCA
At5g07690	*MYB29*	TCCTACAACGGTCGTCTACCA	TTCTTCGGCAGTCCATGCTC
At2g14610	*PR1*	TCTAAGGGTTCACAACCAG	CGCAGCGTAGTTGTAGTTA
At2g38470	*WRKY33*	TCCTGCAGCTCGTGGTAGCGGT	GGCGTAACCAAAAGGCCCGGT
